# Could the Health Decline of Prehistoric California Indians be Related to Exposure to Polycyclic Aromatic Hydrocarbons (PAHs) from Natural Bitumen?

**DOI:** 10.1289/ehp.1103478

**Published:** 2011-05-19

**Authors:** Sebastian K.T.S. Wärmländer, Sabrina B. Sholts, Jon M. Erlandson, Thor Gjerdrum, Roger Westerholm

**Affiliations:** 1 Department of Anthropology, University of California, Santa Barbara, California, USA; 2 Division of Biophysics, Stockholm University, Stockholm, Sweden; 3 Museum of Natural and Cultural History, University of Oregon, Eugene, Oregon, USA; 4 Department of Analytical Chemistry, Stockholm University, Stockholm, Sweden

**Keywords:** bioaccumulation, biomarkers exposure, bone and cartilage, cultural practices, diet and nutrition, environmental epidemiology, indigenous peoples, molecular epidemiology, polycyclic aromatic hydrocarbons, population health

## Abstract

Background: The negative health effects of polycyclic aromatic hydrocarbons (PAHs) are well established for modern human populations but have so far not been studied in prehistoric contexts. PAHs are the main component of fossil bitumen, a naturally occurring material used by past societies such as the Chumash Indians in California as an adhesive, as a waterproofing agent, and for medicinal purposes. The rich archaeological and ethnohistoric record of the coastal Chumash suggests that they were exposed to multiple uptake pathways of bituminous PAHs, including direct contact, fume inhalation, and oral uptake from contaminated water and seafood.

Objectives: We investigated the possibility that PAHs from natural bitumen compromised the health of the prehistoric Chumash Indians in California.

Conclusions: Exposure of the ancient Chumash Indians to toxic PAHs appears to have gradually increased across a period of 7,500 years because of an increased use of bitumen in the Chumash technology, together with a dietary shift toward PAH-contaminated marine food. Skeletal analysis indicates a concurrent population health decline that may be related to PAH uptake. However, establishing such a connection is virtually impossible without knowing the actual exposure levels experienced by these populations. Future methodological research may provide techniques for determining PAH levels in ancient skeletal material, which would open new avenues for research on the health of prehistoric populations and on the long-term effects of human PAH exposure.

In the modern world, our environment abounds in polycyclic aromatic hydrocarbons (PAHs) derived mainly from fossil fuels formed by the anaerobic decomposition of dead organisms over millions of years. The lipophilic PAHs, which chemically consist of two or more condensed aromatic benzene rings and occur in a large number of isomers, are readily taken up by the human body and distributed to different organs and tissues, including the fetus. Significant health problems such as cancer, altered hormone levels, damage to internal organs including the nervous system, and deficiencies in important nutrients such as vitamin A have been associated with exposure to liquid and atmospheric PAHs (Boström et al. 2002; Li et al. 2003; Mastrangelo et al. 1996; Westerholm et al. 1988, 2001). Such exposure can originate from gasoline and diesel combustion, fossil fuel processing, road paving, roofing, and tobacco smoking. PAH exposure has also been associated with reproductive and developmental impairments, including reduced birth length and head circumference in children of women exposed to PAHs during pregnancy (Choi et al. 2006; Perera et al. 1998; Polańska et al. 2010). Occasionally, extraordinary events such as major oil spills result in drastic PAH exposure, which may have severe consequences for human and animal life (Hicken et al. 2011; Wells et al. 1995).

In the ancient world, our ancestors encountered PAHs mostly in the form of fossil bitumen, or asphaltum. Fossil bitumen occurs in geological strata all over the planet. In petroleum-rich areas such as California, Mexico, and the Near East, certain geological formations allow bitumen to seep spontaneously to the surface of the earth. Because of its adhesive and water-repellent properties, bitumen was collected from natural seeps by early human populations and used for a variety of purposes such as sealant for containers and watercraft, as glue for fixing arrowheads and spear points to their shafts, as mortar for constructing roads and houses, and as mastic in the manufacture of art objects such as the famous Sumerian “Standard of Ur” (Connan 1999; Wendt and Lu 2006). Bitumen was also applied to the skin for medicinal purposes and during ritual practices and was used extensively in ancient Egyptian mummification processes (Harrell and Lewan 2002).

Despite the well-documented health risks of PAH exposure in modern populations, we are not aware of any studies addressing potential health impacts of PAH exposure in ancient environments. In this commentary, we discuss the possibility that PAHs from natural bitumen adversely affected the health of the prehistoric Chumash Indians on the Channel Islands in California. Specifically, we examine temporal changes in stature and head size to see if they correlate with archaeological evidence for increasing PAH exposure over time.

## The Channel Islands, the Chumash, and Bitumen

**Figure 1:**
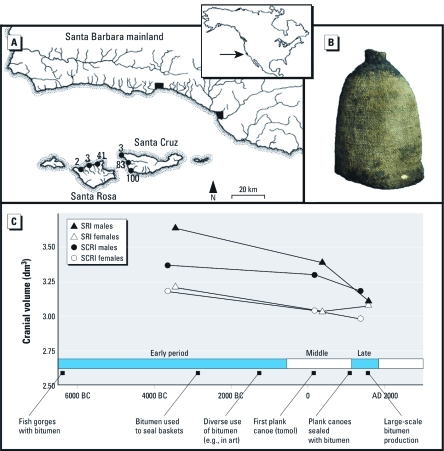
(*A*) Map showing the excavation sites (archaeological sites SCRI-3, -83, and -100; SRI-2, -3, and -41) at Santa Cruz and Santa Rosa Islands, California. (*B*) A bitumen-coated water-bottle basket. Photo courtesy of the Santa Barbara Museum of Natural History. (*C*) Average cranial volumes for Early, Middle, and Late period (King 1990) Santa Rosa Island (SRI) males and females and Santa Cruz Island (SCRI) males and females.

From Point Dume to Point Conception, the Santa Barbara Channel separates the California coast from the four northern Channel Islands of Anacapa, Santa Cruz, Santa Rosa, and San Miguel (Figure 1A). The nutrient-rich waters of this region provide abundant marine resources (Schoenherr et al. 1999), and archaeological evidence shows that humans have occupied the islands and adjacent mainland for at least 13,000 years (Erlandson et al. 2011). The post-Pleistocene sequence of human occupation is generally divided into the Early (6500–600 bc), Middle (600 bc–ad 1150), and Late (ad 1150–1782) periods (King 1990). During these periods, human populations increased in size and density, and their reliance on marine resources, especially fish, intensified (Erlandson et al. 2011; Kennett 2005; Rick et al. 2001). Population aggregation facilitated social differentiation and political centralization, culminating in the complex hunter-gatherer society of the Chumash Indians, first encountered by European explorers in the 16th century (King 1990).

The Santa Barbara Channel region is also one of the world’s most prolific areas of natural hydrocarbon seepage (Allen et al. 1970). Large submarine seeps produce chunks of soft bitumen (“tar balls”) that frequently wash ashore on island and mainland beaches (Hostettler et al. 2004), and there are numerous terrestrial oil seeps from which the native Chumash collected raw bitumen. On San Miguel Island, bitumen occurs in man-made objects between 10,000 and 7,500 years old (Vellanoweth et al. 2003), but becomes more prevalent with the development of bitumen-sealed water-bottle baskets about 5,000 years ago (Braje et al. 2005). These water bottles were waterproofed by swirling pulverized bitumen and hot pebbles inside the basket until the interior surfaces were sealed with melted bitumen (Rogers 1929), a traditional technique that persisted into the 19th century ad (Figure 1B).

**Figure 2:**
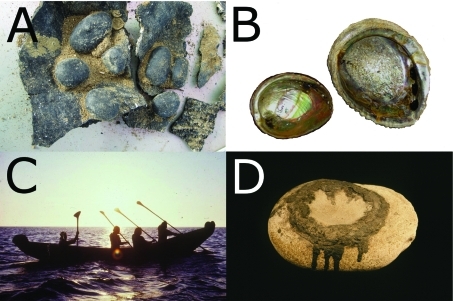
(*A*) Archaeological fragments of a bitumen-coated water-bottle basket, with tarring pebbles still stuck to the interior of the bottom. Photo by J.M.E. (*B*) Abalone shells with their breathing holes plugged with bitumen, possibly used as food containers. (*C*) A modern reconstruction of the bitumen-sealed tomol plank canoe. (*D*) Hopper mortar with residues of the bitumen used to adhere the hopper basket. Photos in *B–D* are courtesy of the Santa Barbara Museum of Natural History.

Bitumen use increased further when the bitumen-sealed plank canoe (“tomol”) was developed around 2,000 years ago (Gamble 2002). When constructing a tomol, chunks of hard bitumen were broken up with pounding stones, mixed with pine pitch and red ochre, and boiled until the substance reached proper consistency (Hudson et al. 1978). As it facilitated cross-channel trade and wide-ranging pelagic fishing, the oceangoing tomol became a cornerstone of the coastal Chumash economy. Consequently, bitumen became a staple commodity among the Chumash, with cakes of the material “always kept on hand for immediate use” (Gutman 1979) and traded all along the channel shores (Hudson et al. 1978). The archaeological evidence for bitumen use in the Santa Barbara Channel region is summarized in Table 1 and Figure 2.

**Table 1:** A brief chronology describing the prehistoric use of bitumen in the Santa Barbara Channel region, California, USA.

Time	Bitumen use	Reference
ad 1000–1500		Over 650 tarring pebbles at coastal mainland settlement (Pitas Point site)		Gamble 1983
ad 1000–1500		Leaf-shaped point replaced with concave base form (abandoned use of asphaltum in hafting)		Gamble 1983
ad 1100		Earliest examples of bitumen caulking and canoe plugs (Simo’mo site)		Gamble 2002
ad 500		Introduction of bow and arrow (bitumen used in attaching leaf-shaped points to arrow shafts)		Glassow et al. 2007
ad 1–500		Plank canoe comes into use		Gamble 2002
1500 bc		Asphaltum in many art objects found in burials		Erlandson and Rick 2002
2000 bc–ad 1		More extensive and diverse use of bitumen		Glassow et al. 2007
2000 bc–ad 1		Transition to contracting stemmed points (with bitumen used to attach point and shaft)		Erlandson 1997
				Glassow et al. 2007
3180–2540 bc		Bitumen basketry impression fragments and tarring pebbles		Braje et al. 2005
8340–6530 bc		Bone bipoints (fish gorges) with evidence of bitumen where they were tied to a fishing line slightly offset from the center		Rick et al. 2001
10000 bc		Earliest Paleocoastal use of mussels and other filter-feeding intertidal mollusks		Erlandson et al. 2011

*Skeletal and bitumen analysis.* Over a century of archaeological research in the Santa Barbara Channel region has produced a detailed record of human demography, technology, and health throughout the past 7,500 years, including a vast database of skeletal populations excavated from island and coastal mainland cemeteries. To determine temporal variation in cranial size among ancient Channel Islands inhabitants, we examined 269 adult crania (135 males and 134 females) from the Early, Middle, and Late period (King 1990) burials on Santa Cruz and Santa Rosa Islands (archaeological sites SCRI-3, -83, and -100; SRI-2, -3, and -41, as shown in Figure 1A), currently housed at the Santa Barbara Museum of Natural History, the Natural History Museum in London, and the Hearst Museum at the University of California, Berkeley. Neighboring populations could not be studied due to insufficient quantities of well-preserved cranial remains from Early period mainland sites and from the San Miguel and Anacapa Islands in general. Dental eruption and wear allowed us to identify adult crania (Buikstra and Ubelaker 1994), and sex was determined from cranial traits (Buikstra and Ubelaker 1994) and from postcranial bones when present (Lambert 1994). Because of the fragile state of some crania, we performed measurements via 3-dimensional (3D) models of the crania created with a portable NextEngine 3D laser scanner (NextEngine, Inc., Malibu, CA, USA), following previously described protocols (Sholts et al. 2010b). For each cranium, we measured standard linear distances of glabello-occipital length, basio-bregmatic height, and maximum cranial breadth (Howells 1973) [see Supplemental Material, Figure 1 (http://dx.doi.org/10.1289/ehp.1103478)]. Multiplying these three distances yields a single volumetric value proportional to the size of the cranial vault (Olivier 1969).

The mean cranial volumes for Early, Middle, and Late period males and females from Santa Cruz and Santa Rosa Islands are shown in Figure 1C. The Santa Cruz Island populations display statistically significant monotonic trends of reduced cranial size between the Early and Late periods, decreasing from 3.37 to 3.18 dm^3^ (*p* = 0.0075) for males and from 3.18 to 2.98 dm^3^ (*p* = 0.016) for females (Figure 1C). For Santa Rosa Island, the male crania show a statistically significant Early-to-Late-period decrease from 3.64 to 3.11 dm^3^ (*p* < 0.0001), whereas the female crania display a statistically significant Early-to-Middle-period size decrease from 3.21 to 3.03 dm^3^ (*p* = 0.031), followed by a slight rebound to 3.08 dm^3^ in the Late period.

Natural bitumen was collected from Simonton Cove (sample I), East Cuyler Harbor (sample II), and Crook Point (sample III) on San Miguel Island (Figure 1A), and from the La Brea Tar Pits in Los Angeles (sample IV). To characterize their chemical composition, one milligram of each sample was subjected to an extraction step with toluene using an ASE 200 (Dionex Corporation, Sunnyvale, CA, USA), cleaned using silica solid phase extraction cartridges, and analyzed with gas chromatography/mass spectrometry using previously described procedures (Bergvall and Westerholm 2008; Westerholm et al. 2001). Prior to extraction, we added internal deuterated PAH standards to each sample in the form of phenanthrene D10, pyrene D10, benzo(*a*)pyrene-D12, and bb-binaphthyl.

A total of 44 unique PAHs were identified in the four bitumen samples [see Supplemental Material, Table 1 (http://dx.doi.org/10.1289/ehp.1103478)]. The PAH levels were higher in the La Brea Tar Pits sample (total PAH content, 2,531 ng/mg) than in the three tar ball samples from San Miguel Island beaches (total PAH contents in the range of 50–110 ng/mg). This finding suggests that some PAHs were dissolved in the ocean or lost to off-gassing as the bitumen dried and hardened onshore. Data were obtained for PAHs with molecular weights in the range of 166–300 g/mol, and it is clear that the samples contained known carcinogens such as phenanthrene, fluoranthene, and pyrene—compounds that are on the 2009 priority pollutant list maintained by the U.S. Environmental Protection Agency (EPA) according to the Clean Water Act (U.S. EPA 2009).

## Discussion

Our chemical analysis establishes the presence of numerous toxic PAHs in natural bitumen from seeps in the Santa Barbara Channel region. Did this constitute an environmental health risk for the native Chumash populations?

Previous research has shown numerous adverse health effects related to PAH exposure in marine organisms that live and feed in the Santa Barbara Channel. Sea urchins and starfish have exhibited developmental impairments and reduced embryonic size as a result of PAH uptake (Allen 1971; Davis et al. 1981; Spies and Davis 1982; Stuermer et al. 1981), and wild fish (rainbow surfperch) have been found to display a range of sublethal effects including inflammation, liver tumors, exocrine pancreas atrophy, and hyperplasia and cartilage dysplasia in the gills (Spies et al. 1996). The PAH levels in bile of the examined fish were 110 μg/g naphthalene equivalents and 140 μg/g phenanthrene equivalents, yielding a tolerable daily human intake of maximum 15 g of such fish [assuming 80 kg body weight and a recommended tolerable daily oral intake of 20 μg naphthalene per kilo bodyweight (Wakefield 2008)]. Increased DNA damage, reduced estradiol levels, and elevated hepatic CYP1A expression were found in hornyhead turbots exposed to bituminous sediment from Coal Oil Point outside Santa Barbara (Roy et al. 2003), a site where offshore marine seeps leak oil at a rate of > 10,000 L/day (Allen et al. 1970). Mussels (*Mytilus* spp.), an important food source for Channel Islanders, also have been shown to readily absorb PAHs into their tissue, although the mussels themselves are only slightly affected by hydrocarbons (Shaw et al. 1988).

The PAHs present in the marine life in the Santa Barbara Channel consequently represent one of several possible sources of PAH uptake for the Channel Islands populations. As PAHs accumulate along the food chain, higher trophic-level predators such as sharks and humans may exhibit the highest PAH levels. During the Middle period, the coastal Chumash shifted their dietary emphasis from terrestrial plants and shellfish to mostly fish (Glassow 1993; Walker and Erlandson 1986), which may have substantially increased their oral intake of PAHs. Moreover, the native Chumash were also exposed to PAHs through their cultural uses of bitumen. PAH uptake through direct contact took place whenever bitumen was gathered, worked, chewed like gum, or applied to the body for ritual or medicinal purposes, such as casts for broken bones or poultices for sore joints. Food items, especially fatty foods, would have absorbed the lipophilic PAHs when stored or served in containers lined with bitumen, such as abalone shells with bitumen-plugged holes (Figure 2B). The development of the bitumen-sealed baskets in the Early period compounded the oral uptake of PAHs, as water stored in bitumen-lined containers would have dissolved at least some of the lighter PAHs such as anthracene and phenanthrene. In addition, PAH-rich fumes would have been inhaled when bitumen was heated or boiled for constructing baskets or tomol canoes, especially after the invention of the tomol in the Middle period. Thus, the ancient Chumash were exposed to a number of possible PAH uptake pathways, and intensified use of bitumen in the Chumash technology together with an increased reliance on near-shore marine fish probably increased PAH exposure over time.

It is difficult to identify the human biological ramifications, if any, of this increasing PAH exposure. Most adverse health outcomes associated with PAH uptake, such as cancer and liver damage, are usually not observable in human skeletal remains and would have varied between individuals. Overall decreases in life span might be expected, but reconstructing mortality profiles for the ancient Channel Island populations is difficult. Not only are age-at-death estimations from skeletal remains relatively imprecise, but elderly individuals are likely to be underrepresented in ancient skeletal assemblages, as accelerated loss of bone calcium due to old age facilitates more rapid bone decomposition (Walker et al. 1988).

PAH exposure has, however, been associated with reduced stature and head circumference in newborn babies of mothers exposed to PAHs during pregnancy (Choi et al. 2006; Perera et al. 1998), and our skeletal analysis demonstrates a statistically significant decrease in cranial size over time among 269 males and females from the Channel Islands (Figure 1C). These results are consistent with previous observations of reduced femur length over time among 149 individuals from the same region, indicating a stature reduction of about 10 cm during the past 7,500 years in Channel Islands populations (Lambert 1993, 1994). The larger number of crania than femora available in museum collections, which allows for more reliable statistics, can be attributed to biased archaeological practices during the late 19th and early 20th centuries, when crania were frequently the only skeletal element collected from burials. As both femur length and cranial size correlate with stature (Işcan 2005; Ryan and Bidmos 2007), our results further substantiate a pattern of stature decrease among Channel Islands populations between the Early and the Late periods. Osteological evidence in the form of rare tooth transpositions has shown that some Early period Channel Islanders suffered from inbreeding (Nelson 1992; Sholts et al. 2010a), a condition that typically prevents people from reaching their full genetic potential for stature (Mange 1964). As inbreeding declined during the Middle period due to increased cross-channel migration (Sholts et al. 2010a), overall population stature would have been expected to rise. The observed stature decline therefore suggests overriding effects from other factors, and because a correlation with increased PAH exposure is observed, the growth impairment may have been partly caused by PAH uptake. The increased cranial size in adult females on Santa Rosa Island between the Middle and Late periods (Figure 1C) is an interesting deviation from the general pattern, which could be related to dietary differences between the sexes, as fishing may have been a predominantly male activity in the Late period (Walker and Hollimon 1989).

Declining health between the Early and Late periods among Santa Barbara Channel populations is further suggested by increased rates of skeletal indicators of disease and stress, such as periosteal lesions, cribra orbitalia, and tooth enamel hypoplasias (Lambert 1993, 1994, 2004; Walker 1986, 1993; Walker and Erlandson 1986). Unfortunately the biomolecular causes behind these bony ailments are unclear (Ortner 2003; Walker et al. 2009) and their increased frequencies over time among Channel Islands populations have so far not been fully explained. Sustained drought conditions between ad 450 and 1300 (Kennett and Kennett 2000) would have reduced terrestrial food and water availability during this time, supporting explanations of Middle period health decline related to nutritional deficiency, climatic change, poor sanitation, and prevalent infectious disease due to population density growth (Lambert 1994). To cope with these conditions, drinking water was most likely stored in larger quantities for prolonged periods of time (Gamble 2005), possibly increasing its PAH contamination. Thus, similar to the stature decline, the higher frequencies of the above conditions could partly be explained by environmental factors such as PAH exposure, but without knowing the actual PAH uptake levels of the ancient Channel Islanders, this connection is tentative.

Because levels of PAH uptake for ancient populations can be reliably determined only from skeletal material, if at all, important future research tasks are to investigate the extent to which PAHs accumulate in bone collagen and to develop techniques for measuring PAH levels in bony tissue. Previous studies on ancient environmental health risks have focused mainly on heavy metal toxicity, such as lead poisoning during the Roman Era (Hernberg 2000), largely because techniques to measure heavy metal content in skeletal material have been readily available. Thus, developing techniques for PAH measurements in the skeleton would open new research avenues, allowing us to better understand the health of specific ancient populations such as the Chumash and their predecessors. Furthermore, such measurement techniques would make it possible to use archaeological material to investigate environmental changes that occur slowly across centuries or millennia, thereby providing useful complements to the controlled and comparative studies on PAH exposure typically carried out for modern populations.

## Summary and Conclusions

The rich archaeological and ethnohistoric records of the native populations of the Santa Barbara Channel Islands provide a rare and valuable opportunity to investigate potential health effects of bituminous PAH exposure in the ancient world. Our results show that natural bitumen in the Santa Barbara Channel region contains numerous PAHs toxic to humans. Coastal Chumash Indians were increasingly exposed to these PAHs as cultural uses of bitumen and consumption of PAH-contaminated water and marine foods intensified over time. Our skeletal analysis revealed a decrease in cranial size in both male and female adults on the northern Channel Islands across a time period of roughly 7,500 years, consistent with a previously suggested decrease in population stature. These trends may be related to increased bituminous PAH exposure, as earlier research has shown that human PAH uptake can compromise fetal growth and development. However, formally establishing such a connection is virtually impossible without knowing the actual levels of exposure experienced by past populations. Because PAH exposure continues to pose a health problem in the modern world as well, we argue the importance of developing measurement techniques that will allow determination of PAH levels in archaeological skeletal material. Such measurements can provide new and important perspectives on prehistoric human health and on the long-term effects of modern human PAH exposure.

## Supplemental Material

(152 KB) PDF
