# Supplementary material for: Could the Health Decline of Prehistoric California Indians be Related to Exposure to Polycyclic Aromatic Hydrocarbons (PAHs) from Natural Bitumen?
Source: Environ Health Perspect. 2011 May 19;119(9):1203–7. doi: 10.1289/ehp.1103478 (PMC3230405; doi:10.1289/ehp.1103478)
Supplement: (152 KB) PDF [file ehp.1103478.s001.pdf]

Supplemental material

Could the Health Decline of Prehistoric California Indians be Related to Exposure to Polycyclic Aromatic Hydrocarbons (PAHs) from Natural Bitumen?

Sebastian K.T.S. Wärmländer, Sabrina B. Sholts, Jon M. Erlandson, Thor Gjerdrum, and Roger Westerholm

Contents

Supplemental Material, Table 1. Concentrations [ng/mg] of polycyclic aromatic hydrocarbons (PAHs) in samples of raw bitumen.

Supplemental Material, Figure 1. Measurements of cranial volume from digital 3D models.

References

Compound	Sample I	Sample II	Sample III	Sample IV	Solubility[a]	Clean Water Act[b]
Fluorene	<0.1	0.1	<0.1	15.3	1980	x
2-Methylfluorene	<0.1	0.9	<0.1	<0.1		
Dibenzothiophene	0.2	4.9	0.5	<0.1		
Phenanthrene	24.6	10.4	13.4	893.7	1290	x
Anthracene	0.3	0.3	<0.1	<0.1	73	x
3-Methylphenanthrene	4.6	2.3	2.8	140.6		
2-Methylphenanthrene	32.9	15.0	17.6	832.2		
2-Methylanthracene	<0.1	<0.1	<0.1	1.0	39	
9-Methylphenanthrene	8.7	2.5	5.1	135.0		
1-Methylphenanthrene	7.5	1.8	3.0	79.9	269	
9-Methylanthracene	<0.1	<0.1	<0.1	<0.1	261	
2-Phenylnaphthalene	0.8	1.1	<0.1	29.1		
3,6-Dimethylphenanthrene	1.1	<0.1	<0.1	<0.1		
3,9-Dimethylphenanthrene	12.4	4.4	7.3	189.7		
Fluoranthene	0.6	0.1	0.3	12.0	260	x
Pyrene	0.7	0.3	0.9	18.8	135	x
9,10-Dimethylanthracene	<0.1	<0.1	<0.1	<0.1	56	
1-Methylfluoranthene	<0.1	<0.1	<0.1	<0.1		
Benz(a)fluorine	<0.1	<0.1	<0.1	22.3	45	
Retene	6.1	1.9	5.3	113.1		
Benz(b)fluorine	0.6	<0.1	<0.1	3.9	2	
2-Methylpyrene	<0.1	<0.1	<0.1	<0.1		
4-Methylpyrene	0.9	<0.1	<0.1	19.7		
1-Methylpyrene	0.4	<0.1	<0.1	15.0		
Benzo(ghi)fluoranthene	<0.1	<0.1	<0.1	<0.1		
Benzo(c)phenanthrene	<0.1	<0.1	<0.1	<0.1		
Benzo(b)naphto(1,2-d)thiophene	<0.1	<0.1	<0.1	9.8		
Cyclopenta(cd)pyrene	<0.1	<0.1	<0.1	<0.1		
Benzo(a)anthracene	1.2	<0.1	0.7	<0.1	14	x
Chrysene	1.1	<0.1	<0.1	<0.1	2	x
3-Methylchrysene	1.2	<0.1	<0.1	<0.1		
2-Methylchrysene	<0.1	<0.1	<0.1	<0.1		
6-Methylchrysene	<0.1	<0.1	<0.1	<0.1		
1-Methylchrysene	<0.1	<0.1	<0.1	<0.1		
Benzo(b)fluoranthene	2.2	3.0	<0.1	<0.1		x
Benzo(k)fluoranthene	<0.1	1.1	<0.1	<0.1		
Benzo(e)pyrene	0.4	<0.1	<0.1	<0.1	5	
Benzo(a)pyrene	<0.1	<0.1	<0.1	<0.1	0.05	x
Perylene	<0.1	<0.1	<0.1	<0.1	0.4	
Indeno(1,2,3-cd)fluoranthene	<0.1	<0.1	<0.1	<0.1		
Indeno(1,2,3-cd)pyrene	<0.1	<0.1	<0.1	<0.1		x
Dibenz(a,h)anthracene	<0.1	<0.1	<0.1	<0.1		x
Benzo(ghi)perylene	<0.1	<0.1	<0.1	<0.1	0.3	x
Coronene	<0.1	<0.1	<0.1	<0.1	0.1	
Sum PAH ng/mg	108.6	50.1	57.0	2531.3		

[a]Solubility in water (µg/liter), values taken from reference (Lee et al. 1981).
[b]Identified on the US EPA 2009 priority pollutant list, according to the Clean Water Act (USEPA 2009).

Supplemental Material, Table 1. Concentrations [ng/mg] of polycyclic aromatic hydrocarbons (PAHs) in samples of raw bitumen (I=Simonton Cove, II=East Cuyler, III=Crook Point, IV= La Brea Tar Pits, Los Angeles).

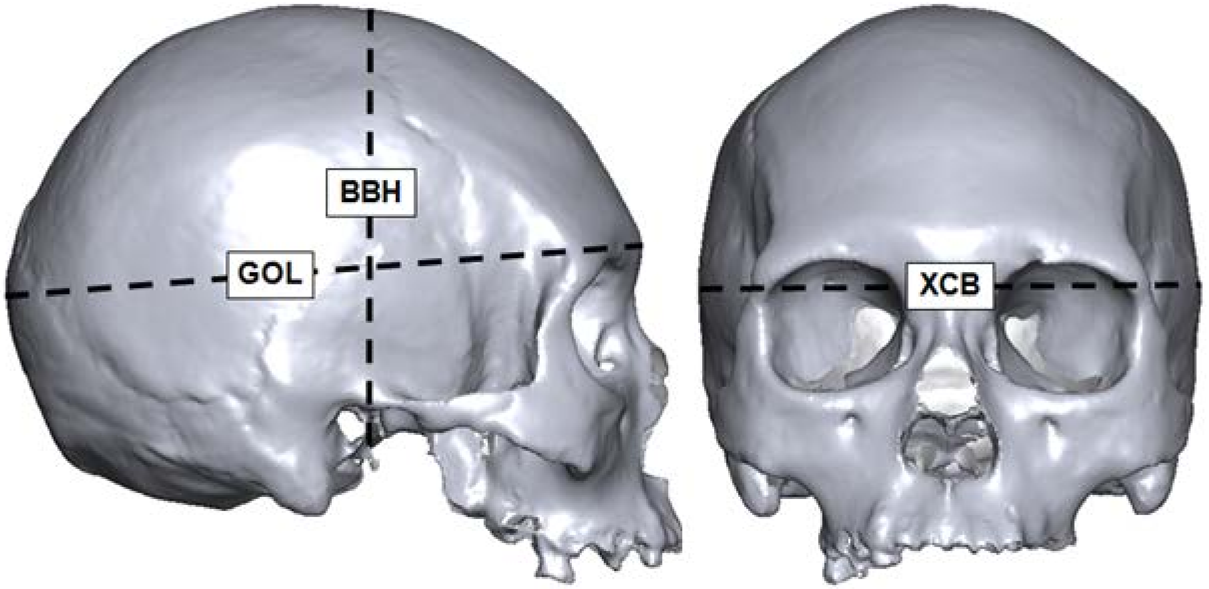

Supplemental Material, Figure 1. Cranial volumes were calculated from digital 3D models by multiplying three linear distances between standardized cranial landmarks, i.e., glabello-occipital length (GOL), basio-bregmatic height (BBH), and maximum cranial breadth (XCB). The landmarks involved have been described by Howells (1973). Olivier (1969) has shown that multiplication of these three linear distances yields a volumetric value that is proportional to the size of the cranial vault. It is in principle possible to obtain the volume of a 3D model as such (Sholts et al. 2010b), but only if the cranium is intact. Hence, the method of measuring three linear distances is better suited for archaeological crania which often display various kinds of damage.

References

Howells WW. 1973. Cranial variation in man: a study by multivariate analysis of patterns of difference among recent human populations. Cambridge, MA: Harvard University Press.

Lee ML, Novotny MV, Bartle KD. 1981. Analytical chemistry of polycyclic aromatic compounds. New York: Academic Press.

Olivier G. 1969. Practical anthropology. Springfield: C. C. Thomas.

Sholts SB, Wärmländer SKTS, Flores LM, Miller KWP, Walker PL. 2010b. Variation in the Measurement of Cranial Volume and Surface Area Using 3D Laser Scanning Technology. J Forensic Sci 55:871-876.

USEPA. 2009. Priority pollutants list. Available: http://www.epa.gov/NE/npdes/permits/generic/prioritypollutants.pdf [accessed July 14, 2010.
